# The group of primitive Pythagorean triples over Gaussian integers

**DOI:** 10.1016/j.heliyon.2023.e16747

**Published:** 2023-06-01

**Authors:** Attawut Wongpradit, Ekkasit Sangwisut

**Affiliations:** a Department of Mathematics and Statistics, Faculty of Science and Technology, Thammasat University (Rangsit Center), Pathum Thani 12120, Thailand; b Department of Mathematics and Statistics, Faculty of Science, Thaksin University, Phattalung 93110, Thailand

**Keywords:** Pythagorean triple, Gaussian integers, Free abelian group

## Abstract

A group structure of the set of primitive Pythagorean triples over Gaussian integers is investigated. In addition, we show that it is a free abelian group. Base on the study, the construction and enumeration of the primitive Pythagorean triples having the same hypotenuse are established.

## 1 Introduction

The Pythagorean theorem states that the summation of the squares on each leg of a right triangle is equal to the square of the hypotenuse, or equivalently, $a^{2}+b^{2}=c^{2}$ where *c* is the length of the hypotenuse while *a* and *b* represent the length of the legs of a right triangle.

**Figure:**
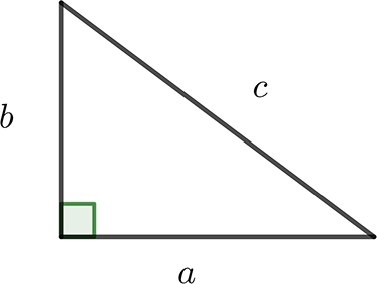

An ordered triple of integers (a,b,c) satisfying the Pythagorean theorem is said to be a **Pythagorean triple (PT)**. Furthermore, the PT is called **primitive**, if gcd⁡(a,b,c)=1. PTs have been extensively studied due to their nice algebraic structure and potential applications to cryptography and graph theory [1], [2]. Let (a,b,c) be a primitive Pythagorean triple (PPT) be such that a,c are odd and *b* is even. One of the well-known methods that characterize the PPTs is the Euclid formula [3, Theorem 12.1]:

$$\left(a,b,c)=(u^{2}-v^{2},2uv,u^{2}+v^{2}\right)$$

for positive integers u,v such that u>v,gcd⁡(u,v)=1 and u,v have different parity. In 1962, the question “How many PPTs have the same hypotenuse?” was asked by W. Sierpinski [4]. After 22 years, E. J. Eckert in [5] answered the question by using a group structure of the set of PPTs of natural numbers and (1,0,1) with a binary operation

(1) (a,b,c)⊗(d,e,f)=(ad−be,ae+bd,cf).

In [6], the number of PPTs of a fixed odd leg has been determined by S. Jitman and E. Sangwisut. They consider the set of PPTs of integers and defined a binary operation as follows:

(a,b,c)⁎(d,e,f)=(ad,bf+ce,be+cf).

Then the set of PPTs of integers with ⁎ is a free abelian group. It is of natural interest to study the number of PPTs over Gaussian integers of a fixed hypotenuse or leg. In [7], the unique factorization of PPTs over the Gaussian integers is studied lead to the number of PPTs over Gaussian integers of a given leg. Therefore, the number of PPTs over Gaussian integers of a given hypotenuse is the only remaining case. In this paper, we focus on the set of PPTs (a,b,c) where a,c are odd and *b* is even with respect to an operation defined in Eq. (1). So, it is a free abelian group. Based on this characterization, the enumeration and construction of all PPTs having the same fixed hypotenuse are established.

## 2 Gaussian integers

The definition and essential properties of Gaussian integers are recalled in this section. For a more introduction to Gaussian integers, the read is referred to [8, Chapter 6] and [9], [10].

Let Z be the set of integers. The Gaussian integers denoted by Z[i] is the set of all complex numbers x+yi where x,y∈Z and $i^{2}=-1$, i.e.,

Z[i]={x+yi|x,y∈Z}.

Namely, the Gaussian integers are the set of complex numbers which both real part and imaginary part are integers. Under usual addition and multiplication, the Gaussian integers form a ring which is a subring of the ring of complex number. Moreover, it is a unique factorization domain.

Let z=x+yi be an element in Z[i]. The **conjugate** of *z* denoted by $\bar{z}$ is the Gaussian integer x−yi. The **norm** of *z* is defined to be $N(z)=z\cdot \bar{z}=(x+yi)(x-yi)=x^{2}+y^{2}$. Clearly, N(z)≥0 for all z∈Z[i]. A **unit** in the ring Z[i] is any element in Z that has a multiplicative inverse in Z[i]. Thus, the units of Z[i] are only 1,−1,i and −*i*. Two Gaussian integers *z* and $z^{\prime }$ are said to be **associated** if $z=\epsilon z^{\prime }$ for some unit *ϵ*. In other words, *z* and $z^{\prime }$ divide each other in Z[i]. A **Gaussian prime** *p* of Z[i] is a nonzero, nonunit such that p=α⋅β implies *α* is a unit or *β* is a unit. Equivalently, if α|p then *α* is a unit or *α* associated to *p*. For given nonzero Gaussian integers *z* and $z^{\prime }$ a **greatest common divisor**, denoted by $\gcd(z,z^{\prime })$, is a divisor with the largest norm. The greatest common divisor of two Gaussian integers is not unique unlike the greatest common divisor of two integers. There are four associated greatest common divisor over Gaussian integers.

In Z, an integer w∈Z is even integer if 2|w and *w* is odd integer for otherwise. Similarly in the Gaussian integer 1+i is chosen to defined odd and even by let z∈Z[i], then *z* is **even** if (1+i)|z and it is **odd** for otherwise (see [10, p. 107]). In order to avoid any confusion, “even integer” and “odd integer” are defined over Z, while “odd” and “even” are defined over Z[i]. The following theorem is useful for determining odd and even in Z[i].

Theorem 1 *The Gaussian integer,*x+yi*is even if and only if*x≡y(mod2)*.*

Proof Let x+yi be even. Then $\frac{x+yi}{1+i}=\frac{\left(x+yi)(1-i\right)}{\left(1+i)(1-i\right)}=\frac{(x+y)+(-x+y)i}{2}$ is a Gaussian integer if x≡y(mod2).

Conversely, we assume that x≡y(mod2). We will show that x+yi is even. Consider $\frac{x+yi}{1+i}\cdot \frac{1-i}{1-i}=\frac{(x+y)+(-x+y)i}{2}\in Z[i]$, since x≡y(mod2). □

We recall the prime factorization in Z[i] analogous to Z.

Theorem 2 *A Gaussian integer c can be written uniquely as a product of Gaussian primes up to order and multiplication of primes by units*

$$c={\left(1+i\right)}^{r_{0}}p_{1}^{r_{1}}\ldots p_{k}^{r_{k}},$$

*where*$p_{j}$*is an odd Gaussian prime, the exponents*$r_{1},\ldots ,r_{k}$*are positive integers and*$r_{0},k$*are nonnegative integers.*

In the case where *c* is odd, the next corollary can be obtained from Theorem 2.

Corollary 3 *Let c be an odd Gaussian integer. Then it can be factored as*

$$c=p_{1}^{r_{1}}\ldots p_{k}^{r_{k}},$$

*where*$p_{j}$*is an odd Gaussian prime, the exponents*$r_{1},\ldots ,r_{k}$*are positive integers and k is a nonnegative integer.*

## 3 PPTs over Gaussian integers

In this section, we define PTs over Gaussian integers similar to PTs over integers. An ordered triple (a,b,c) where a,b and *c* are Gaussian integers satisfying the Pythagorean theorem is called a **PT**. In addition, a PT with gcd⁡(a,b,c)=1 is called **primitive**. For a given PPT (a,b,c), there are exactly two elements in the set {a,b,c} such that they are odd and the rest element is an even [7, p. 5], e.g., (3,4,5),(5,4i,3),(5i,3,4i) and (5,−3i,4). For convenience, *a* and *b* are said to be **leg** and *c* is called **hypotenuse**. In the entire paper, we focus on PPTs (a,b,c) where a,c are odd and *b* is even. The following lemmas are useful.

Lemma 4 **Lemma 2.8 of [7]**

(a,b,c)*is a PT if and only if*(c,bi,a)*is a PT.*

Lemma 5 *Let*$u=u_{1}+u_{2}i$*and*$v=v_{1}+v_{2}i$*be odd. Then the following hold:*

1. *If*$u_{i}\equiv v_{i}\;(\mathrm{mod}\;2)$*for all*i=1,2*, then*$4|(u^{2}-v^{2})$*.*
2. *If*$u_{1}\equiv v_{2}\;(\mathrm{mod}\;2)$*and*$u_{2}\equiv v_{1}\;(\mathrm{mod}\;2)$*, then*$4|(u^{2}+v^{2})$*.*

Proof For 1., it is obvious that

$$u^{2}-v^{2}=(u+v)(u-v)=\left((u_{1}+v_{1})+(u_{2}+v_{2})i\right)\left((u_{1}-v_{1})+(u_{2}-v_{2})i\right).$$

We have $4|(u^{2}-v^{2})$, since $u_{i}\equiv v_{i}\;(\mathrm{mod}\;2)$ for all i=1,2.

For 2., note that

$$u^{2}+v^{2}=(u+vi)(u-vi)=\left((u_{1}-v_{2})+(u_{2}+v_{1})i\right)\left((u_{1}+v_{2})+(u_{2}-v_{1})i\right).$$

By $u_{1}\equiv v_{2}\;(\mathrm{mod}\;2)$ and $u_{2}\equiv v_{1}\;(\mathrm{mod}\;2)$, we have $4|(u^{2}+v^{2})$. □

We recall the Euclid formula over an arbitrary unique factorization domain given by [11].

Theorem 6 *If*D≠(0)*is a unique factorization domain of characteristic not* 2*, then every PT can be written as the form* $a=\frac{f(u^{2}-v^{2})}{d},\;b=\frac{2fuv}{d}$ *and* $c=\frac{f(u^{2}+v^{2})}{d}$*, where* f,u,v∈D *such that* $d|2,d|(u^{2}\pm v^{2})$ *and* gcd⁡(f,d)=1*.*

When D=Z and the PTs are primitive, we obtain the Euclid formula as well as D=Z[i] and the PTs are primitive, we obtain the following corollary.

Corollary 7 *The PPTs*(a,b,c)*over*Z[i]*are determined by the formulae*$a=u^{2}-v^{2},\;b=2uv$*and*$c=u^{2}+v^{2}$*, where*u,v∈Z[i]*such that*u≢v(mod1+i)*and*gcd⁡(u,v)=1*.*

Proof Let (a,b,c) be a PPT. It follows that a,c are odd and *b* is even. By Theorem 6, *f* is a unit and gcd⁡(u,v)=1 unless (a,b,c) is not primitive. Applying Theorem 6, $a=\frac{u^{2}-v^{2}}{d},\;b=\frac{2uv}{d}$ and $c=\frac{u^{2}+v^{2}}{d}$ such that d|2 and $d|(u^{2}\pm v^{2})$.

Case I*u* and *v* are odd. By Lemma 5, we have $4|(u^{2}-v^{2})$, a contradiction to *a* is odd.

Case II*u* and *v* are even. Then $2|(u^{2}\pm v^{2})$, we choose d=2i. We write $u=(1+i)\overset{ˆ}{u}$ and $v=(1+i)\overset{ˆ}{v}$ for some $\overset{ˆ}{u},\overset{ˆ}{v}\in Z[i]$. Thus, we have

$$a=\frac{u^{2}-v^{2}}{2i}=\frac{{\left(1+i\right)}^{2}({\overset{ˆ}{u}}^{2}-{\overset{ˆ}{v}}^{2})}{2i}={\overset{ˆ}{u}}^{2}-{\overset{ˆ}{v}}^{2},b=\frac{2uv}{2i}={\left(1+i\right)}^{2}\overset{ˆ}{u}\overset{ˆ}{v}=2\overset{ˆ}{u}\overset{ˆ}{v},c=\frac{u^{2}+v^{2}}{2i}=\frac{{\left(1+i\right)}^{2}({\overset{ˆ}{u}}^{2}+{\overset{ˆ}{v}}^{2})}{2i}={\overset{ˆ}{u}}^{2}+{\overset{ˆ}{v}}^{2}.$$

If both $\overset{ˆ}{u}$ and $\overset{ˆ}{v}$ are even (resp., odd), then $1+i|({\overset{ˆ}{u}}^{2}-{\overset{ˆ}{v}}^{2})=a$, a contradiction to *a* is odd.

If $\overset{ˆ}{u}$ is odd and $\overset{ˆ}{v}$ is even (resp., $\overset{ˆ}{u}$ is even and $\overset{ˆ}{v}$ is odd), then *a* and *c* are odd and *b* is even.

Case III*u* is even and *v* is odd. We have that $u^{2}\pm v^{2}$ is odd, which implies d=1. Therefore, $a=u^{2}-v^{2},\;b=2uv$ and $c=u^{2}+v^{2}$, which a,c are odd and *b* is even.

Case IV*u* is odd and *v* is even. This case is similar to case III.

All together, we can conclude that $a=u^{2}-v^{2},\;b=2uv$ and $c=u^{2}+v^{2}$, where gcd⁡(u,v)=1 and u≢v(mod1+i) as desired. □

In the special case $c=p^{k}$ where *p* is an odd Gaussian prime, we derive specific PPTs as follows.

Corollary 8 *Given an odd Gaussian prime power*$p^{k}$*. Hence the PPTs*(a,b,c)*where*$c=p^{k}$*are of the form*$a=\pm \frac{p^{2k}+1}{2},\;b=\pm \frac{p^{2k}-1}{2}i$*and*$c=p^{k}$*, where the sign* ± *are pairwise independent.*

Proof Given a PT $\left(a,b,p^{k}\right)$. By Lemma 4, $\left(p^{k},bi,a\right)$ is a PT as well. Using Corollary 7, we have $p^{k}=u^{2}-v^{2}=(u+v)(u-v)$ and bi=2uv for some u,v in Z[i]. Let $u+v=\epsilon p^{k-\ell }$ and $u-v=\epsilon^{-1}p^{\ell }$ for some unit *ϵ* and 0≤ℓ≤k. If 1≤ℓ≤k−1, then gcd⁡(u,v)≠1. Consequently, (a,b,c) is not primitive. So ℓ=0 or *k*. Then the possible cases are in the following table

**Figure:**
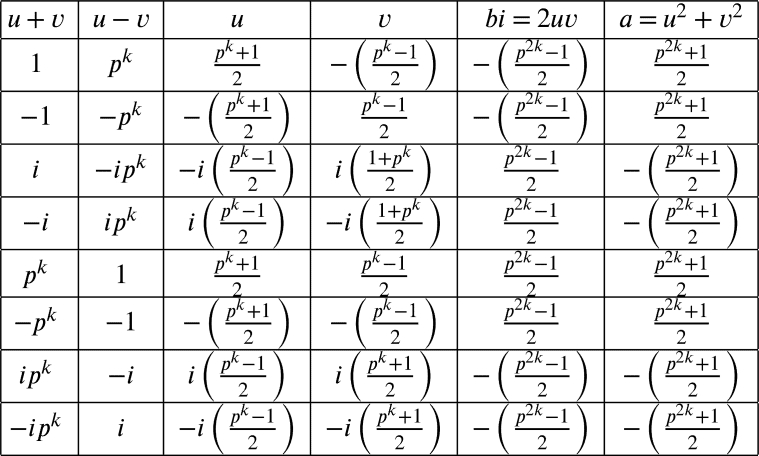

It can be concluded that $(p^{k},bi,a)=\left(p^{k},\pm \frac{p^{2k}-1}{2},\pm \frac{p^{2k}+1}{2}\right)$. Thus, by Lemma 4, we have $(a,b,p^{k})=\left(\pm \frac{p^{2k}+1}{2},\pm \frac{p^{2k}-1}{2}i,p^{k}\right)$ as desired. □

## 4 The group (P,⊗) and PPTs with odd hypotenuse

Let P denote the set of PTs. Let *R* be an equivalence relation on P defined by R={((a,b,c),(d,e,f))∈P×P|(a,b,c)=k(d,e,f) for some k∈Q[i]∖{0}}. The equivalence class of (a,b,c) with respect to *R* is denoted by [a,b,c]. Let P/R denote the family of all equivalence classes on P.

Theorem 9 *The set*P/R*and the binary operation defined by*

[a,b,c]⊗[d,e,f]=[ad−be,ae+bd,cf]

*for all*[a,b,c],[d,e,f]∈P/R*, form an abelian group.*

Proof First we prove that ⊗ is well-defined. Let [ka,kb,kc],[ℓd,ℓe,ℓf]∈P/R for some k,ℓ∈Q[i]∖{0}. Consider [ka,kb,kc]⊗[ℓd,ℓe,ℓf]=[kℓad−kℓbe,kℓae+kℓbd,kℓcf]=[ad−be,ae+bd,cf]. So ⊗ is well-defined.

We examine

$${\left(ad-be\right)}^{2}+{\left(ae+bd\right)}^{2}=a^{2}d^{2}-2adbe+b^{2}e^{2}+a^{2}e^{2}+2abde+b^{2}d^{2}=a^{2}d^{2}+b^{2}e^{2}+a^{2}e^{2}+b^{2}d^{2}=a^{2}(d^{2}+e^{2})+b^{2}(d^{2}+e^{2})=(a^{2}+b^{2})(d^{2}+e^{2})=c^{2}f^{2}.$$

Therefore, [ad−be,ae+bd,cf]∈P/R which implies the set P/R is closed with respect to ⊗.

For [a,b,c],[d,e,f]∈P/R, we get

[a,b,c]⊗[d,e,f]=[ad−be,ae+bd,cf]=[da−eb,db+ea,fc]=[d,e,f]⊗[a,b,c].

Hence, *R* is commutative. Therefore, it suffices to show that there are identity and inverse elements only on one-sided.

Given elements [a,b,c],[d,e,f] and [x,y,z] in P/R. Thus

([a,b,c]⊗[d,e,f])⊗[x,y,z]=[ad−be,ae+bd,cf]⊗[x,y,z]=[(ad−be)x−(ae+bd)y,(ad−be)y+(ae+bd)x,(cf)z]=[adx−bex−aey−bdy,ady−bey+aex+bdx,cfz]=[a(dx−ey)−b(dy+ex),a(dy+ex)+b(dx−ey),c(fz)]=[a,b,c]⊗[dx−ey,dy+ex,fz]=[a,b,c]⊗([d,e,f]⊗[x,y,z]).

Hence, the associative law holds.

Let [a,b,c]∈P/R. Consider

[1,0,1]⊗[a,b,c]=[a+0,b+0,c]=[a,b,c]

Then [1,0,1] is an identity element with respect to ⊗.

For any choice of [a,b,c]∈P/R,

$$[a,b,c]⊗[a,-b,c]=[a^{2}+b^{2},-ab+ba,c^{2}]=[c^{2},0,c^{2}]=[1,0,1],$$

which implies [a,−b,c] is an inverse of [a,b,c] relative to ⊗. Altogether, we can conclude that the group of (P/R,⊗) is abelian. □

In order to prove our main result Theorem 12, we need the following lemmas.

Lemma 10 *Given Gaussian integers*a,b,d*and e. For an odd Gaussian prime p such that*$abde≢0\;(\mathrm{mod}\;p^{2})$*and*$d^{2}+e^{2}\equiv a^{2}+b^{2}\equiv 0\;(\mathrm{mod}\;p^{2})$*. Therefore, only one of these statements holds.*

1. $ad+be\equiv bd-ae\equiv 0\;(\mathrm{mod}\;p^{2})$*.*
2. $ad-be\equiv bd+ae\equiv 0\;(\mathrm{mod}\;p^{2})$*.*

Proof Consider $\left(ad+be)(ad-be)=a^{2}d^{2}-b^{2}e^{2}=a^{2}d^{2}+b^{2}d^{2}-b^{2}d^{2}-b^{2}e^{2}=d^{2}(a^{2}+b^{2})-b^{2}(d^{2}+e^{2})\equiv 0\;(\mathrm{mod}\;p^{2}\right)$. Suppose that $p^{2}|(ad+be)$ and $p^{2}|(ad-be)$. Hence, we obtain $p^{2}|4abde$, a contradiction. Therefore $p^{2}|(ad+be)$ or $p^{2}|(ad-be)$ but not both. Hence, we have 2 cases as follows:

**Case I**$p^{2}|(ad+be)$. Thus, $p^{4}|{\left(ad+be\right)}^{2}$ and consider

$${\left(ad+be\right)}^{2}+{\left(bd-ae\right)}^{2}=(d^{2}+e^{2})(a^{2}+b^{2})\equiv 0\;(\mathrm{mod}\;p^{4})$$

We have $p^{4}|{\left(bd-ae\right)}^{2}$ which implies $p^{2}|(bd-ae)$.

**Case II**$p^{2}|(ad-be)$. Hence, $p^{4}|{\left(ad-be\right)}^{2}$ and we have that

$${\left(ad-be\right)}^{2}+{\left(bd+ae\right)}^{2}=(d^{2}+e^{2})(a^{2}+b^{2})\equiv 0\;(\mathrm{mod}\;p^{4})$$

Consequently, $p^{4}|{\left(bd+ae\right)}^{2}$ and $p^{2}|(bd+ae)$ as desired. □

Lemma 11 *For an arbitrary PPT*[a,b,c]*and an odd Gaussian prime p such that*$p^{r}|c$*and*$\gcd(p,\frac{c}{p^{r}})=1$*. We write*$q=\frac{c}{p^{r}}$*. Let*$\left[\pm d,\pm e,p^{r}\right]$*be PPTs. Then we have the following primitive representations.*

1. $[a,b,c]⊗[d,-e,p^{r}]=\left[\frac{ad+be}{p^{2r}},\frac{bd-ae}{p^{2r}},q\right]$
2. $[a,b,c]⊗[-d,e,p^{r}]=\left[\frac{-ad-be}{p^{2r}},\frac{-bd+ae}{p^{2r}},q\right]$
3. $[a,b,c]⊗[d,e,p^{r}]=\left[\frac{ad-be}{p^{2r}},\frac{bd+ae}{p^{2r}},q\right]$
4. $[a,b,c]⊗[-d,-e,p^{r}]=\left[\frac{-ad+be}{p^{2r}},\frac{-bd-ae}{p^{2r}},q\right]$

Proof In 1., we have $\left[a,b,c]⊗[d,-e,p^{r}]=[ad+be,bd-ae,p^{r}c\right]$. Next, we will show that $\left[\frac{ad+be}{p^{2r}},\frac{bd-ae}{p^{2r}},q\right]$ is a PPT. Suppose that $s|\gcd\left(\frac{ad+be}{p^{2r}},\frac{bd-ae}{p^{2r}},q\right)$. Then

$$b=\frac{b(d^{2}+e^{2})}{p^{2r}}=\frac{e(ad+be)}{p^{2r}}+\frac{d(bd-ae)}{p^{2r}},$$

which implies s|b. This is a contradiction to gcd⁡(b,c)=1. The proof of 2. follows similarly.

In 3., we obtain $\left[a,b,c]⊗[d,e,p^{r}]=[ad-be,bd+ae,p^{2r}q\right]$. Claim that $\left[\frac{ad-be}{p^{2r}},\frac{bd+ae}{p^{2r}},q\right]$ is a PPT. Assume that $\gcd\left(\frac{ad-be}{p^{2r}},\frac{bd+ae}{p^{2r}},q\right)=s$. Hence

$$b=\frac{b(d^{2}+e^{2})}{p^{2r}}=\frac{-e(ad-be)}{p^{2r}}+\frac{d(bd+ae)}{p^{2r}}.$$

We have that s|b. This will contradict the assumption that [a,b,c] is primitive. In 4. can be proved in the same manner. □

Theorem 12 *The set*P/R*with a binary operation* ⊗ *is a free abelian group with a basis is the set of PPTs of odd Gaussian prime hypotenuse. Precisely, each PPT having hypotenuse c can be established as*

(2) $$[a,b,c]=r_{1}\left[\pm \frac{p_{1}^{2}+1}{2},\pm \frac{p_{1}^{2}-1}{2}i,p_{1}\right]⊗\cdots ⊗r_{k}\left[\pm \frac{p_{k}^{2}+1}{2},\pm \frac{p_{k}^{2}-1}{2}i,p_{k}\right],$$

*where* $c=p_{1}^{r_{1}}\ldots p_{k}^{r_{k}}$*, k and* $r_{j}$ *are positive integers, the number* $p_{1},p_{2},\ldots ,p_{k}$ *are different odd Gaussian primes moreover the signs* ± *are pairwise independent.*

Proof We use mathematical induction on *k*. Base case, let $c=p_{1}^{r_{1}}$. By applying Corollary 8, we have

$$[a,b,p_{1}^{r_{1}}]=\left[\pm \frac{p_{1}^{2r_{1}}+1}{2},\pm \frac{p_{1}^{2r_{1}}-1}{2}i,p_{1}^{r_{1}}\right]=r_{1}\left[\pm \frac{p_{1}^{2}+1}{2},\pm \frac{p_{1}^{2}-1}{2}i,p_{1}\right].$$

For the induction step, let k>1 be a fixed integer. We suppose that Eq. (2) is true for all integers less than *k*. Let $c=p_{1}^{r_{1}}\ldots p_{k}^{r_{k}}$ and write $c=p_{1}^{r_{1}}q$ where $q=p_{2}^{r_{2}}\ldots p_{k}^{r_{k}}$. Since $p_{1}^{r_{1}}$ is an odd Gaussian prime power, $\left[\pm \frac{p_{1}^{2r_{1}}+1}{2},\pm \frac{p_{1}^{2r_{1}}-1}{2}i,p_{1}^{r_{1}}\right]$ are the PPTs by Corollary 8. We write $d=\frac{p_{1}^{2r_{1}}+1}{2}$ and $e=\frac{p_{1}^{2r_{1}}-1}{2}i$. As [a,b,c] and $\left[\pm d,\pm e,p_{1}^{r_{1}}\right]$ are PPTs, $\pm abde≢0\;(\mathrm{mod}\;p_{1}^{2r_{1}})$ and $a^{2}+b^{2}\equiv d^{2}+e^{2}\equiv 0\;(\mathrm{mod}\;p_{1}^{2r_{1}})$. Applying Lemma 10, there are two cases.

**Case I**$ad+be\equiv bd-ae\equiv 0\;(\mathrm{mod}\;p_{1}^{2r_{1}})$. Therefore, we obtain

$$[a,b,c]=[1,0,1]⊗[a,b,c]=\left([d,e,p_{1}^{r_{1}}]⊗[d,-e,p_{1}^{r_{1}}]\right)⊗[a,b,c]=[d,e,p_{1}^{r_{1}}]⊗\left([d,-e,p_{1}^{r_{1}}]⊗[a,b,c]\right)=[d,e,p_{1}^{r_{1}}]⊗[ad+be,bd-ae,p_{1}^{r_{1}}c]=[d,e,p_{1}^{r_{1}}]⊗\left[\frac{ad+be}{p_{1}^{2r_{1}}},\frac{bd-ae}{p_{1}^{2r_{1}}},q\right],\text{ by Lemma }\text{11}\text{ and }c=p_{1}^{r_{1}}q\;,=\left[\frac{p_{1}^{2r_{1}}+1}{2},\frac{p_{1}^{2r_{1}}-1}{2}i,p_{1}^{r_{1}}\right]⊗\left[\frac{ad+be}{p_{1}^{2r_{1}}},\frac{bd-ae}{p_{1}^{2r_{1}}},q\right]\text{ by Corollary }\text{8},=r_{1}\left[\frac{p_{1}^{2}+1}{2},\frac{p_{1}^{2}-1}{2}i,p_{1}\right]⊗r_{2}\left[\pm \frac{p_{2}^{2}+1}{2},\pm \frac{p_{2}^{2}-1}{2}i,p_{2}\right]⊗\cdots ⊗r_{k}\left[\pm \frac{p_{k}^{2}+1}{2},\pm \frac{p_{k}^{2}-1}{2}i,p_{k}\right],\text{ by induction hypothesis,}$$

and

$$[a,b,c]=[1,0,1]⊗[a,b,c]=\left([-d,-e,p_{1}^{r_{1}}]⊗[-d,e,p_{1}^{r_{1}}]\right)⊗[a,b,c]=[-d,-e,p_{1}^{r_{1}}]⊗\left([-d,e,p_{1}^{r_{1}}]⊗[a,b,c]\right)=[-d,-e,p_{1}^{r_{1}}]⊗[-ad-be,-bd+ae,p_{1}^{r_{1}}c]=[-d,-e,p_{1}^{r_{1}}]⊗\left[\frac{-ad-be}{p_{1}^{2r_{1}}},\frac{-bd+ae}{p_{1}^{2r_{1}}},q\right],\text{ by Lemma }\text{11}\text{ and }c=p_{1}^{r_{1}}q\;,=\left[-\left(\frac{p_{1}^{2r_{1}}+1}{2}\right),-\left(\frac{p_{1}^{2r_{1}}-1}{2}\right)i,p_{1}^{r_{1}}\right]⊗\left[\frac{-ad-be}{p_{1}^{2r_{1}}},\frac{-bd+ae}{p_{1}^{2r_{1}}},q\right]\text{ by Corollary }\text{8},=r_{1}\left[-\left(\frac{p_{1}^{2}+1}{2}\right),-\left(\frac{p_{1}^{2}-1}{2}\right)i,p_{1}\right]⊗r_{2}\left[\pm \frac{p_{2}^{2}+1}{2},\pm \frac{p_{2}^{2}-1}{2}i,p_{2}\right]\;\;\;⊗\cdots ⊗r_{k}\left[\pm \frac{p_{k}^{2}+1}{2},\pm \frac{p_{k}^{2}-1}{2}i,p_{k}\right],\text{ by induction hypothesis.}$$

**Case II**$ad-be\equiv bd+ae\equiv 0\;(\mathrm{mod}\;p_{1}^{2r_{1}})$. Then, we have

$$[a,b,c]=[1,0,1]⊗[a,b,c]=\left([d,-e,p_{1}^{r_{1}}]⊗[d,e,p_{1}^{r_{1}}]\right)⊗[a,b,c]=[d,-e,p_{1}^{r_{1}}]⊗\left([d,e,p_{1}^{r_{1}}]⊗[a,b,c]\right)=[d,e,p_{1}^{r_{1}}]⊗[ad-be,bd+ae,p_{1}^{r_{1}}c]=[d,-e,p_{1}^{r_{1}}]⊗\left[\frac{ad-be}{p_{1}^{2r_{1}}},\frac{bd+ae}{p_{1}^{2r_{1}}},q\right],\text{ by Lemma }\text{11}\text{ and }c=p_{1}^{r_{1}}q\;,=\left[\frac{p_{1}^{2r_{1}}+1}{2},-\left(\frac{p_{1}^{2r_{1}}-1}{2}\right)i,p_{1}^{r_{1}}\right]⊗\left[\frac{ad-be}{p_{1}^{r_{1}}},\frac{bd+ae}{p_{1}^{2r_{1}}},q\right]\text{ by Corollary }\text{8},=r_{1}\left[\frac{p_{1}^{2}+1}{2},-\left(\frac{p_{1}^{2}-1}{2}\right)i,p_{1}\right]⊗r_{2}\left[\pm \frac{p_{2}^{2}+1}{2},\pm \frac{p_{2}^{2}-1}{2}i,p_{2}\right]⊗\cdots ⊗r_{k}\left[\pm \frac{p_{k}^{2}+1}{2},\pm \frac{p_{k}^{2}-1}{2}i,p_{k}\right],\text{ by induction hypothesis,}$$

and

$$[a,b,c]=[1,0,1]⊗[a,b,c]=\left([-d,e,p_{1}^{r_{1}}]⊗[-d,-e,p_{1}^{r_{1}}]\right)⊗[a,b,c]=[-d,e,p_{1}^{r_{1}}]⊗\left([-d,-e,p_{1}^{r_{1}}]⊗[a,b,c]\right)=[-d,e,p_{1}^{r_{1}}]⊗[-ad+be,-bd-ae,p_{1}^{r_{1}}c]=[-d,e,p_{1}^{r_{1}}]⊗\left[\frac{-ad+be}{p_{1}^{2r_{1}}},\frac{-bd-ae}{p_{1}^{2r_{1}}},q\right],\text{ by Lemma }\text{11}\text{ and }c=p_{1}^{r_{1}}q\;,=\left[-\left(\frac{p_{1}^{2r_{1}}+1}{2}\right),\frac{p_{1}^{2r_{1}}-1}{2}i,p_{1}^{r_{1}}\right]⊗\left[\frac{-ad+be}{p_{1}^{2r_{1}}},\frac{-bd-ae}{p_{1}^{2r_{1}}},q\right]\text{ by Corollary }\text{8},=r_{1}\left[-\left(\frac{p_{1}^{2}+1}{2}\right),\frac{p_{1}^{2}-1}{2}i,p_{1}\right]⊗r_{2}\left[\pm \frac{p_{2}^{2}+1}{2},\pm \frac{p_{2}^{2}-1}{2}i,p_{2}\right]\;\;\;⊗\cdots ⊗r_{k}\left[\pm \frac{p_{k}^{2}+1}{2},\pm \frac{p_{k}^{2}-1}{2}i,p_{k}\right],\text{ by induction hypothesis.}$$

All together, it can be concluded that

$$[a,b,c]=r_{1}\left[\pm \frac{p_{1}^{2}+1}{2},\pm \frac{p_{1}^{2}-1}{2}i,p_{1}\right]⊗\cdots ⊗r_{k}\left[\pm \frac{p_{k}^{2}+1}{2},\pm \frac{p_{k}^{2}-1}{2}i,p_{k}\right],$$

where the signs ± are pairwise independent. □

Example 1 Let c=5=(1+2i)(1−2i). The PPTs with hypotenuse 5 are of the forms

$$[a,b,5]=\left[\pm \frac{{\left(1+2i\right)}^{2}+1}{2},\pm \frac{{\left(1+2i\right)}^{2}-1}{2}i,1+2i\right]⊗\left[\pm \frac{{\left(1-2i\right)}^{2}+1}{2},\pm \frac{{\left(1-2i\right)}^{2}-1}{2}i,1-2i\right]=\left[\pm (-1+2i),\pm (-2-2i),1+2i\right]⊗\left[\pm (-1-2i),\pm (2-2i),1-2i\right]$$

which are listed in the following table.

**Figure:**
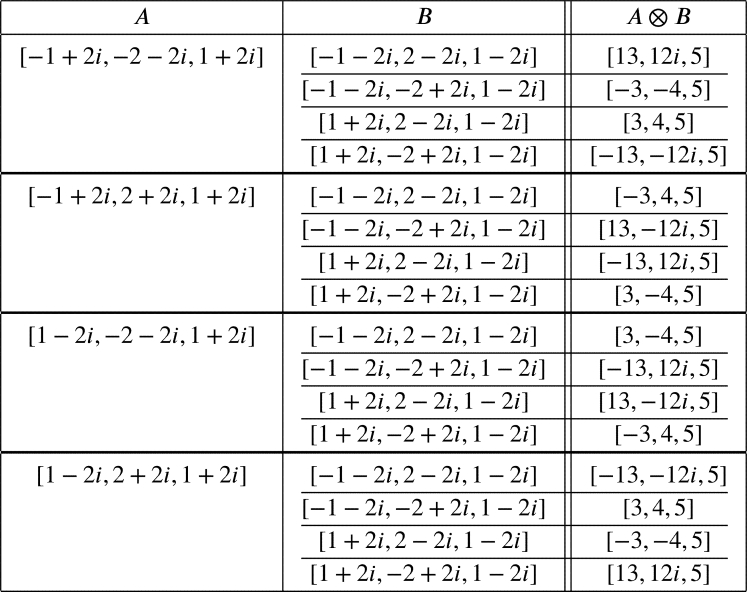

Let [a,b,c],[d,e,f] be PPTs. Since [a,b,c]⊗[d,e,f]=[−a,−b,c]⊗[−d,−e,f], there are $\frac{2^{4}}{2}=8$ distinct PPTs with hypotenuse 5.

## 5 Conclusion

The main goal of the current study was to find the PPTs over Gaussian integers having the same odd hypotenuse. A group structure of the set of PPTs over Gaussian integers is investigated. The construction of PPTs over Gaussian integers on a fixed odd hypotenuse was conducted via a group structure.

The scope of this study was limited in terms of the PPTs where the hypotenuses are odd. The question raised by this study is how to construct and enumerate the PPTs over Gaussian integers on a fixed even hypotenuse.

## Funding statement

Attawut Wongpradit was supported by 10.13039/501100005790Thammasat University [TUFT 59/2565].

## CRediT authorship contribution statement

Attawut Wongpradit: Conceived and designed the experiments; Performed the experiments; Wrote the paper.

Ekkasit Sangwisut: Analyzed and interpreted the data; Contributed reagents, materials, analysis tools or data; Wrote the paper.

## Declaration of Competing Interest

The authors declare that they have no known competing financial interests or personal relationships that could have appeared to influence the work reported in this paper.

## Data Availability

No data was used for the research described in the article.
